# The Clinical and Diagnostic Characterization of 6q24-Related Transient Neonatal Diabetes Mellitus: A Polish Pediatric Cohort Study

**DOI:** 10.3390/biomedicines13102492

**Published:** 2025-10-13

**Authors:** Michał Pietrusiński, Julia Grzybowska-Adamowicz, Tomasz Płoszaj, Sebastian Skoczylas, Maciej Borowiec, Katarzyna Piekarska, Bogda Skowrońska, Małgorzata Wajda-Cuszlag, Artur Mazur, Agnieszka Zmysłowska

**Affiliations:** 1 Department of Clinical Genetics, Medical University of Lodz, 92-213 Lodz, Poland; julia.grzybowska-adamowicz@umed.lodz.pl (J.G.-A.); tomasz.ploszaj@umed.lodz.pl (T.P.); sebastian.skoczylas@umed.lodz.pl (S.S.); maciej.borowiec@umed.lodz.pl (M.B.); katarzyna.piekarska2@student.umed.lodz.pl (K.P.); agnieszka.zmyslowska@umed.lodz.pl (A.Z.); 2 Department of Pediatric Diabetes, Auxology, and Obesity, Poznan University of Medical Sciences, 61-701 Poznan, Poland; bskowron@ump.edu.pl; 3 Department of Endocrinology and Diabetology, The Children’s Memorial Health Institute, 04-730 Warsaw, Poland; malgwa@tlen.pl; 4 Clinic of Pediatric Endocrinology and Pediatric Diabetology, Medical College of Rzeszow University, 35-959 Rzeszow, Poland; armazur@ur.edu.pl

**Keywords:** TNDM, 6q24, MS-MLPA, methylation

## Abstract

**Background/Objectives:** Transient neonatal diabetes mellitus (TNDM) is a form of neonatal diabetes mellitus (NDM) arising in the first weeks of life and remitting in infancy. Epigenetic aberrations at the imprinted 6q24 locus (overexpression of *PLAGL1*/*HYMAI*) are the most common causes of TNDM. The aim of this study was a retrospective clinical and genetic analysis of a Polish pediatric cohort, emphasizing the role of methylation-specific MLPA (MS-MLPA) in the diagnosis of TNDM. **Methods:** We conducted a retrospective analysis of the medical records of 22 patients with diabetes diagnosed at 1 year of age. The molecular studies included an analysis of the NDM gene panel by a targeted NGS and MS-MLPA for the 6q24 imprinting region. **Results:** 6q24-TNDM was confirmed in five patients, with a median age of diabetes remission of 4 months (IQR: 3–6 months). The MS-MLPA identified paternal UPD6 or isolated maternal hypomethylation of *PLAGL1* in three patients, and two had a paternal 6q24 duplication. **Conclusions:** In our group, changes in the 6q24 region were confirmed in 22.7% of NDM patients, indicating the usefulness of the MS-MLPA technique in the diagnosis and detection of imprinting defects. We acknowledge key limitations, including diagnostic delays and incomplete parental testing, which precluded trio-based confirmation of paternal UPD6 versus epimutation in some cases; future diagnostic workflows should incorporate an early trio-based SNP array or STR confirmation. A methylation analysis should be included early in the NDM genetic diagnosis process to provide genetic counseling and monitor patients for diabetes recurrence.

## 1. Introduction

Monogenic diabetes (MD) is a rare form of diabetes, usually resulting from a single gene mutation. MD can manifest during the neonatal period—as neonatal diabetes mellitus (NDM) or, more commonly, before the age of 35–45 as maturity-onset diabetes of the young (MODY) or syndromic forms of monogenic diabetes [1]. NDM is defined as the onset of severe hyperglycemia within the first six months of life, although it can, rarely, manifest during the second six months of a child’s life [2]. It is clinically heterogeneous, often monogenic, and exists in two primary forms: permanent (PNDM) and transient (TNDM). Its clinical manifestations commonly include intrauterine growth restriction (IUGR), failure to thrive, polyuria, and severe dehydration [2]. Depending on the underlying genetic cause, some individuals may also present with congenital anomalies, like an umbilical hernia or developmental delay [3]. NDM is rare, with an estimated incidence of 1 in 90,000 to 260,000 live births [4], and is evenly distributed between PNDM and TNDM cases. Multi-locus imprinting disturbances (MLIDs), such as those caused by recessive *ZFP57* mutations, can compound the phenotype (e.g., congenital heart defects, hypotonia) in some cases [5].

TNDM typically manifests within the first week of life due to impaired insulin secretion, with spontaneous remission occurring by 18 months of age in most cases [5]. However, approximately 50% of affected individuals will experience a relapse during adolescence or early adulthood. The majority of TNDM cases (60–70%) are attributed to overexpression of paternally expressed imprinted genes *PLAGL1* and *HYMAI* on chromosome 6q24, resulting from paternally inherited duplications, paternal uniparental disomy of chromosome 6 (UPD6), or hypomethylation of the maternal *PLAGL1*/*HYMAI* imprinting control region, leading to inappropriate maternal allele expression [6]. Although these alterations are distinct entities with different etiologies and recurrence risks, they double the dose of *PLAGL1*/*HYMAI* transcripts, disrupting beta-cell development or survival and causing neonatal hyperglycemia [7]. Genetic testing has become the essential standard for the precise diagnosis and management of NDM. According to the consensus guidelines, next-generation sequencing (NGS) of known monogenic diabetes genes is recommended as a first-line test in infants diagnosed within 6 months of age [6]. Such panels typically include *KCNJ11*, *ABCC8*, *INS*, and other NDM genes, but they do not investigate the imprinting status. Since 6q24-TNDM results from imprinting changes and copy number variants, standard sequencing panels would not detect these defects [8]. Instead, methylation-specific assays, such as MS-MLPA, are required to detect abnormal methylation and copy number variations in 6q24 [9].

To our knowledge, this is the first national 6q24-TNDM case series from Poland (and Central/Eastern Europe), addressing a regional data gap and adding geographic diversity to the global literature on imprinting disorders and neonatal diabetes. The aim of this study was to clinically and genetically analyze patients with suspected neonatal diabetes using both MS-MLPA and targeted NGS for detecting epigenetic changes.

## 2. Materials and Methods

**Figure 1:**
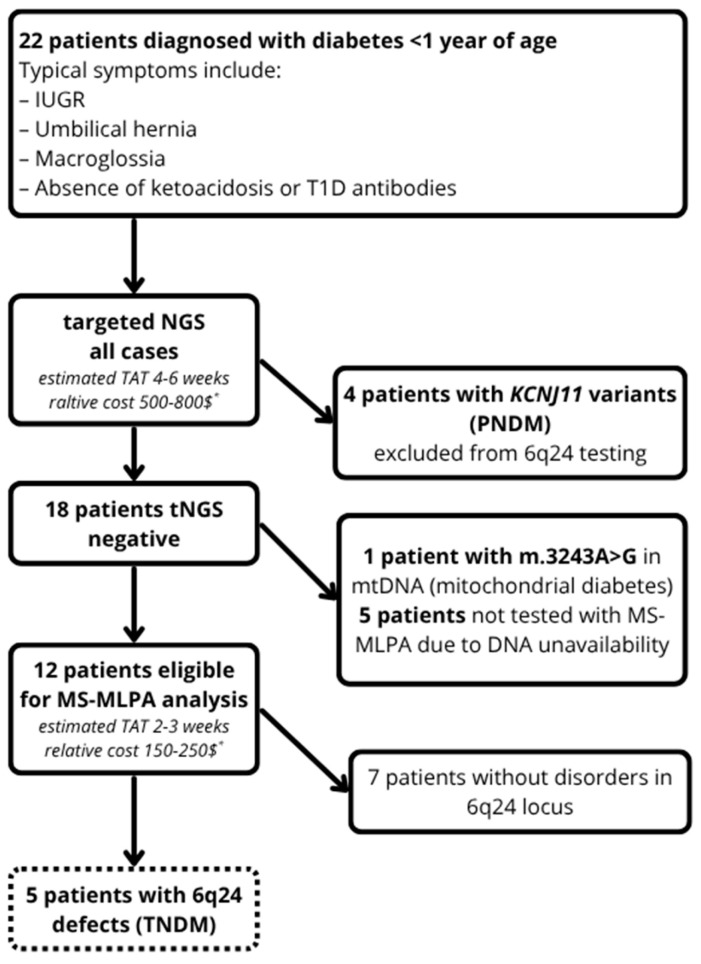
The flowchart of the conducted study for neonatal diabetes, emphasizing integrated genetic testing. Diagnostic flow summary: 22 total → 4 solved by tNGS (*KCNJ11*) → 18 eligible for 6q24 testing → 12 tested by MS-MLPA (5 no DNA; 1 mtDNA diagnosis) → 5 positive (yield among tested: 41.7%, 95% CI: 15–72%; overall yield: 22.7%, 95% CI: 8–45%). TAT—turnaround time. *—country-dependent.

The study retrospectively analyzed the medical records of 22 patients (11 females, 11 males) who were referred to the Outpatient Clinic for Rare Diseases in Children and Adolescents and Diabetogenetics in Lodz, Poland, between 2017 and 2024 for diabetes diagnosed before the age of one (Figure 1). While classical 6q24-TNDM usually manifests within the first week of life, we included all infants with an onset <12 months in accordance with international recommendations that any infant diagnosed with diabetes before 12 months should be considered for genetic testing [10]. In our cohort, none of the 22 infants had onset between 6 and 12 months; all presented within the first 2 months of life (latest was P4 at day 14).

Written informed consent for genetic testing and long-term follow-up was obtained from all parents/guardians of participants and/or their relatives, per local regulations. DNA from peripheral blood was extracted from each patient and their available family members (parents and siblings when possible) (Maxwell, Promega, Madison, WI, USA). First, targeted next-generation sequencing (tNGS, Illumina, San Diego, CA, USA) panel for NDM was performed (*ABCC8*, *AIRE*, *CNOT1*, *CTLA4*, *EIF2AK3*, *EIF2B1*, *FOXP3*, *GATA4*, *GATA6*, *GCK*, *GLIS3*, *HYMAI*, *IER3IP1*, *IL2RA*, *INS*, *ITCH*, *KCNJ11*, *KCNMA1*, *LRBA*, *MNX1*, *NEUROD1*, *NEUROG3*, *NKX2-2*, *ONECUT1*, *PAX6*, *PLAGL1*, *SLC19A2*, *SLC2A2*, *STAT1*, *STAT3*, *STAT5B*, *WFS1*, *YIPF5*, *ZFP57*, and *SLC19A2*) and covered coding regions and exon–intron boundaries (sequencing coverage 99% at ≥30× across target regions; variants classified as likely pathogenic/pathogenic according to ACMG guidelines). Subsequently, MS-MLPA (Methylation-specific Multiplex Ligation-dependent Probe Amplification; SALSA MLPA probemix ME033-A1, MRC-Holland, Amsterdam, the Netherlands) analysis was performed in 12 of 18 tNGS-negative patients. One was diagnosed with mitochondrial diabetes (m.3243A>G variant in mtDNA); remaining five were not tested due to DNA unavailability (Figure 1). Thus, testing pathway was as follows: all 22 underwent tNGS first → 4 solved by *KCNJ11* variants (excluded from 6q24 testing) → 18 were eligible for MS-MLPA → 12 tested by MS-MLPA (5 lacked DNA; 1 had mtDNA diagnosis).

The MS-MLPA findings were interpreted by comparing the sample probe peak heights to the reference samples. A decreased methylation index (ratio of methylated to unmethylated signal) indicated hypomethylation. An increased total probe dosage indicated duplication. The complete loss of the maternal methylated probe (and doubling of the paternal probe signal) indicated paternal uniparental disomy (UPD6). The underlying genetic mechanism (UPD6, duplication, or isolated hypomethylation) was determined from the pattern of methylation and the copy number results [11]. The methylation dosage ratios were calculated using MRC-Holland’s Coffalyser software, v.240129.1959. The affected patients with 6q24 hypomethylation had methylation indices ranging from 0.01 to 0.05 (normal ~0.50), indicating a near-complete loss of methylation. The analytical precision of the MS-MLPA assay was estimated at ±5% for methylation values, based on the probe variability and manufacturer performance specifications. A sufficient number (≥3) of reference samples was included in each MS-MLPA experiment for data normalization. The paternal UPD6 or hypomethylation findings were not confirmed using an SNP array, short tandem repeat (STR) analysis, or parental studies, as parental samples were unavailable. As a result, paternal UPD6 could not be definitively confirmed in some cases, and these were conservatively classified as “hypomethylation/UPD6”; this limitation is acknowledged.

Clinical data were collected on gender, gestational age, birth weight, age of onset of diabetes (in days), insulin dosing regimen at diagnosis, age of spontaneous remission (discontinuation of insulin use for at least 1 month), and any congenital anomalies or comorbidities (including macroglossia, umbilical hernia, and heart defects). The detailed clinical characteristics of the 6q24-TNDM patients are shown in Table 1 (includes birthweight percentiles, maternal diabetes/obstetric history, consanguinity/family history, and footnotes indicating atypical presentations).

For the statistical analysis, the proportions are reported with 95% confidence intervals (CIs) calculated by the exact Clopper–Pearson method. For the skewed variables, we report both the mean ± SD and median (IQR). The diagnostic yields are shown using multiple denominators (tested, eligible, and entire cohort). The STROBE checklist was followed.

## 3. Results

Table 1 summarizes the clinical data and genetic results. The median gestational age was 37 weeks (range: 29–41 weeks; mean: 36.6 ± 5.0). All but one infant (P4) were small for their gestational age (birth weight <3rd centile); the mean birth weight was 1848 g (range: 1050–2450 g, ±594). The average age at diabetes onset was 4.4 days (range: 1–14 days, ±5.1). At diabetes diagnosis, all required insulin therapy. The initial insulin requirements averaged 0.6 U/kg/day (SD: 0.2; median: 0.6 U/kg/day; IQR: 0.5–0.7). None of the patients had diabetic ketoacidosis at the time of diagnosis and the autoantibodies characteristic of autoimmune type 1 diabetes were absent.

Diagnostic yield: Of the 22 infants, 18 were eligible for a 6q24 MS-MLPA after excluding 4 with pathogenic *KCNJ11* variants. Twelve of the eighteen were tested, and five had 6q24 abnormalities: diagnostic yield, 5/12 = 41.7% (95% CI, Clopper–Pearson: 15–72%). For context, this corresponds to 5/18 = 27.8% (95% CI: 10–53%) among the eligible patients, and 5/22 = 22.7% (95% CI: 8–45%) for the entire cohort.

The associated findings included umbilical hernias (P2 and P5); autism and a mild speech delay (P1); ADHD (P4); and an atrial septal defect, increased limb muscle tone, and decreased abdominal muscle tone (P5). Umbilical hernias occurred in 2/5 (40%; 95% CI: ~5–85%); macroglossia was not documented (0/5; 95% CI: 0–45%). There were no other major cardiac malformations or neurological deficits noted in our cohort. The neurodevelopmental features were abstracted retrospectively from the charts (no standardized assessments were performed), and should be interpreted cautiously without causal inference. The additional clinical features are consistent with reports linking 6q24 imprinting defects to neurodevelopmental outcomes, with the exception of P4, who did not have IUGR and showed an atypical phenotype.

All the patients experienced spontaneous remission of diabetes within 2–6 months of life (median: 4 months) and did not take insulin thereafter. The average age at genetic diagnosis was 8.8 years (±10.6; range: 1–26 years, reflecting substantial diagnostic delays). The median age was 4 years, with an IQR of 1.1–12 years. Patient P1 experienced a recurrence of the disease at the age of 12, indicating the need for continuous monitoring. This patient was born at 41 weeks of gestation, with IUGR characteristics and a birth weight of 1950 g. On the second day of life, the patient was diagnosed with diabetes and treated with insulin. The diabetes persisted for 6 months. At the age of 12, hyperglycemia was observed (glycated hemoglobin; HbA1c = 6.4%). Three years later, the patient was diagnosed with diabetes based on an oral glucose tolerance test (0 min 123 mg/dL (6.8 mmol/L); 120 min 203 mg/dL (11.3 mmol/L)). Their HbA1c value was 7.5%, and the antibodies characteristic of type 1 diabetes were negative. The patient was treated with diet and long-acting insulin. At the age of 17, due to inadequate glycemic control (HbA1c = 9.7%), a sulfonylurea derivative was added. An ophthalmological examination revealed no signs of diabetic retinopathy. The NGS testing showed no pathogenic variants in the selected gene panel. The MS-MLPA analysis revealed a duplication of 6q24 on the paternal allele, and the patient was diagnosed with 6q24-TNDM. The molecular testing of the family showed that the patient’s brother also had a paternal 6q24 duplication of the 6q24 region. He was diagnosed with diabetes at the age of 12 based on an HbA1c = 6.6%.

The four remaining patients were developing normally; one child (with paternal 6q24 duplication) had a mild speech delay but no other neurological disorders. However, one patient (P4) was atypical in having a later onset (14 days) and no IUGR despite confirmed paternal 6q24 duplication. This patient, an 11-year-old boy, was referred with suspected neonatal diabetes. Diabetes was diagnosed on his 14th day of life. He was treated with insulin therapy until his 5th month, when remission was noted. Lately, he had had hyperglycemia (fasting glycemia ranges from 70 to 130 mg/dL (3.9–7.2 mmol/L), but during stress or infection can be over 200 mg/dL (11.1 mmol/L)). His HbA1c was 5.3% and islet-related antibodies were negative. In addition, the patient had cryptorchidism (after surgical treatment) and attention deficit hyperactivity disorder (ADHD). The NGS analysis found no pathogenic variants in the selected gene panel. Then, the patient was diagnosed with 6q24-TNDM based on the presence of paternal duplication of 6q24 locus. The array CGH confirmed an ~4.82 Mb duplication at 6q24.1–q24.4 (chr6:139,869,103–144,696,744), including the imprinted genes *PLAGL1* and *HYMAI*. This highlights phenotypic variability even with classic genetic mechanisms.

The targeted NGS panels detected pathogenic variants in the known NDM gene in 4/22 (18.2%) patients. The four positive cases carried *KCNJ11* variants: NM_000525.3: c.149G>A (2 cases), NM_000525.3: c.209A>C, and NM_000525.4: c.601C>T.

Next, an MS-MLPA analysis performed on 12/18 patients revealed abnormalities in locus 6q24 in 5 patients: 2 patients had paternal duplication of the 6q24 region, and another 3 patients had either paternal uniparental disomy of this region or isolated maternal hypomethylation involving the *PLAGL1* gene. The remaining six patients were not tested (five due to DNA unavailability, and in one patient the pathogenic variant m.3243A>G in mtDNA was found), and this is recognized as a limitation of the study.

The MS-MLPA yielded precise diagnostic results in all cases. In patients P1 and P4, the MS-MLPA indicated three copies of the DMR, with two unmethylated (paternal) signals and one methylated (maternal) signal, consistent with a paternal duplication of 6q24 (Patient P4 underwent an array CGH test, revealing a pathogenic duplication in the size of 4.82 Mb on chromosome 6q.24.1q24.4 at the 139869103_144696744 chromosomal position, with overexpression of the *PLAGL1* and *HYMAI* genes.). In patients P2, P3, and P5, the MS-MLPA showed two copies of 6q24 (one maternal, one paternal) by dosage, but either the loss of the maternally methylated probe indicating paternal uniparental disomy (UPD6) of chromosome 6 was found or the maternal allele was largely unmethylated (methylation index approaching 0), indicating isolated hypomethylation of the maternal *PLAGL1* differentially methylated region (DMR). This pattern is compatible with paternal UPD6 or a defect in imprinting on the maternal allele. To investigate the possibility of a multi-locus imprinting defect, the *ZFP57* gene was sequenced in these three cases, but no pathogenic variants were identified. We were unable to perform a parental SNP array or STR confirmation in these cases due to unavailable parental samples (limitation).

Moreover, molecular analyses were also performed for the first-degree relatives of the patients, where available. The MS-MLPA technique performed on nine family members led to the diagnosis of 6q24-TNDM in one person. No other disease-causing variants were identified in any of these nine patients.

## 4. Discussion

Our study focuses on the clinical and genetic characteristics of children with 6q24-related TNDM to highlight the diagnostic value of MS-MLPA and demonstrate how an integrated molecular approach influences clinical decision-making and the assessment of recurrence risk. The MS-MLPA clearly identified the underlying molecular mechanism in nearly 42% (95% CI: 15–72%) of patients referred for testing (22.7% of total cohort), identifying either paternal uniparental disomy of chromosome 6 (UPD6), isolated maternal hypomethylation at locus 6q24, or paternal duplication of the 6q24 region. These findings are consistent with the known distribution of pathogenic mechanisms in 6q24-TNDM cohorts [3]. No patient showed evidence of larger rearrangements at 6q24 or multi-locus imprinting defects. Furthermore, none of the patients presented with features beyond those typical of 6q24-TNDM. In our cohort of patients with 6q24-TNDM, the clinical picture was consistent with the classic phenotype described in the literature [7]. All the patients had diabetes diagnosed within the first 2 weeks of life, required insulin treatment, and achieved spontaneous remission in infancy. Moreover, the presence of an umbilical hernia in 40% of our cases is in accordance with previous reports [12]. Table 2 positions our cohort against international series, showing overall concordance (very early onset, high IUGR/SGA rate), with a lower observed macroglossia frequency (0%) that may reflect true absence or under-reporting in retrospective charts.

At the molecular level, our cohort included all three classic 6q24-TNDM mechanisms. Temple et al. found that patients with paternal UPD6 or multiple imprinting defects often have more birth defects than those with isolated duplications [12]. In our group, the paternal UPD6 or hypomethylation cases had IUGR and feeding problems, but also an umbilical hernia (2 cases) and atrial septal defect, and increased limb muscle tone and decreased abdominal muscle tone (one case). According to the literature, 33–56% of patients with 6q24-TNDM have macroglossia and about 20% experience an umbilical hernia [2,10,14].

Course of illness in P4 could be described as atypical due to lack of IUGR, as majority of patients are born small for gestational age. Additionally, only about 30% are born prematurely [2,10]. Beyond the congenital anomalies, we also observed the following neurodevelopmental findings: autism (P1), ADHD (P4), and a mild speech delay in one duplication case. These align with prior evidence that 6q24 epimutations may be associated with neurodevelopmental issues.

However, the data on neurodevelopmental features came from retrospective clinical records and standardized neurodevelopmental assessments (e.g., Bayley-III/IV, WISC-V, Conners scales) that were not performed on our cohort, and the data were limited to retrospective clinical records. This represents a limitation of the study. We recommend that future prospective studies include formal neurocognitive testing to better define the long-term neurodevelopmental phenotype associated with 6q24 imprinting defects and to guide the appropriate early intervention strategies. Information on parental clinical and molecular data would be most valuable to obtain in future studies.

Similar to our study, a report by Alkorta-Aranburu et al. observed that the MS-MLPA identified all known 6q24 abnormalities, including cases in which a clinical NGS did not identify the cause until an MS-MLPA revealed a 6q24 imprinting defect [8]. This confirms our observations that targeted NGS panels are inadequate for detecting methylation changes or uniparental disomy, and that relying solely on sequencing risks missing or delaying diagnosis of TNDM. Importantly, the mean age at genetic diagnosis in our cohort was 8.8 years, reflecting a substantial delay that may negatively affect family counseling and patient surveillance in terms of diabetes relapses. Referring patients earlier for genetic testing and treating this diagnostic test as a routine procedure for neonatal diabetes could prevent these delays. In practical terms, delayed molecular confirmation can lead to missed opportunities: identifying additional affected family members earlier (e.g., P1’s sibling), adapting appropriate monitoring to the risk of diabetes recurrence during adolescence/pregnancy, and to enable timely referral to a developmental specialist during follow-up.

In addition to the correct diagnosis, identifying 6q24’s cause has important clinical implications. Families can be counseled about the recurrence risk: paternal UPD6 is almost always de novo (recurrence ~0), whereas a paternal 6q24 duplication may recur if the father carries a balanced rearrangement. Likewise, hypomethylation cases may require testing for recessive genes (e.g., ZFP57) that could affect siblings [15]. Missing parental molecular data affects the assessment of the risk of the disease developing in a patient’s siblings. A summary of recurrence risks and recommended genetic counseling actions is presented in Table 3.

In our cohort, genetic counseling was tailored accordingly. The long-term follow-up for these patients will focus on growth monitoring and screening for diabetes relapse, especially during puberty or, for females, pregnancy. In our cohort, two patients (P1 and P4) deviated from the classical 6q24-TNDM phenotype. P1 had a late molecular diagnosis and adolescent recurrence; P4 lacked IUGR and had a later onset (day 14) despite a confirmed 6q24 duplication—highlighting the phenotypic variability and the need for early genetic confirmation even when IUGR is absent. No specific pharmacological therapy (such as sulfonylureas used for *KCNJ11* mutations [16]) is available for the initial phase of 6q24-TNDM-associated diabetes, but recognition of the etiology confirms that standard diabetes management is appropriate and that insulin can often be discontinued once remission is achieved. Insulin remains the standard treatment during the initial hyperglycemic phase. During remission, glycemic control is typically maintained without therapy, although some individuals require ongoing treatment with sulfonylureas, insulin, or both [17]. Counseling summaries for each 6q24 mechanism are provided in Table 3.

The results of the MS-MLPA test also had a direct impact on genetic counseling. For patients P2, P3, and P5 (with paternal UPD6 or hypomethylation), a sporadic origin was presumed, suggesting a negligible recurrence risk in future pregnancies [1]. In cases of isolated hypomethylation, an underlying mutation in an imprinting maintenance gene, such as *ZFP57,* could be considered [15]; however, this was ruled out by the targeted next-generation sequencing (tNGS). Detection of sporadic epimutations is beyond the scope of standard technologies currently applied in diagnostic laboratories. For patients P1 and P4, who carry paternal 6q24 duplications, the possibility of inherited chromosomal rearrangements was considered [18]. Although both fathers were asymptomatic, they were offered genetic counseling and informed of the potential for balanced rearrangements involving the 6q24 locus. By contrast, the targeted NGS alone failed to provide diagnostic information in these cases, as imprinting defects at 6q24 are not identifiable through a sequence analysis alone [8]. Thus, only by combining MS-MLPA with NGS were we able to provide reproducible and precise results for our cohort and establish a definitive molecular diagnosis in all tested cases; however, it must be noted that MS-MLPA alone does not distinguish between epimutation and paternal UPD6 without additional parental or SNP-based data. This highlights the value of incorporating SNP arrays or trio-based STR testing into future diagnostic workflows. Our findings support the existing evidence that a comprehensive testing strategy—including a methylation analysis—is necessary to resolve the majority of neonatal diabetes etiologies [2]. Given these results, we recommend that the genetic evaluation of neonatal diabetes include both sequencing of monogenic diabetes genes and an imprinting analysis for 6q24. A proposed diagnostic workflow is shown in Figure 1. If an infant presents with diabetes before 6 months and has features such as IUGR, umbilical hernia, or macroglossia, a methylation assay (MS-MLPA or equivalent) should be performed early. Rapid MS-MLPA testing can confirm a diagnosis of 6q24-TNDM and spare the patient from unnecessary interventions. In resource-limited settings, access to MS-MLPA could be scaled by centralized referral testing, batched runs to reduce per-sample costs, and clear clinical triggers (IUGR, umbilical hernia, or early hyperglycemia) to prioritize cases. Our findings are largely consistent with the published 6q24-TNDM cohorts from Western Europe, in terms of molecular mechanism distribution and clinical phenotype. However, relatively few studies have described 6q24-TNDM cohorts from non-Western settings. For example, a recent study from Sri Lanka reported on 6q24-related diabetes in a small series of infants, with some atypical presentations and delayed diagnoses due to limited testing access [19]. Our study also has some limitations, including its small sample size and retrospective nature. Selection bias is possible because only 12/18 eligible patients underwent MS-MLPA. Our broad inclusion criterion (<12 months) aligns with guidelines, but could theoretically dilute the specificity relative to a strict neonatal definition; in practice, none of our cases presented after 2 months. Additionally, missing parental samples prevented confirmation of paternal UPD6 versus epimutation in some cases. However, it emphasizes the broader principle that epigenetic disorders require specialized diagnostic testing. The advantages of MS-MLPA—simultaneous copy number and methylation analysis—make it optimal for 6q24-TNDM. Alternative methods, such as a methylation-sensitive PCR or chromosome microarray, can detect some abnormalities, but MS-MLPA is relatively fast and cost-effective for targeted imprinting loci. With the advent of new technologies, future studies may explore genome-wide methylation profiling [20], but for now, MS-MLPA remains a practical and widely available assay for clinical testing [8]. To our knowledge, this is the first Polish cohort with 6q24-related TNDM to be reported. Our national cohort adds geographic diversity to the literature and illustrates practical diagnostic implementation in routine care.

In conclusion, our case series illustrates the classic features of 6q24-associated TNDM and the central role of epigenetic mechanisms in its pathogenesis. We have shown that MS-MLPA should be given its rightful place in the routine diagnosis of neonatal diabetes. An early and accurate molecular diagnosis facilitates personalized follow-up and family counseling for this form of monogenic diabetes.

## 5. Conclusions

The use of MS-MLPA is essential for the diagnosis of 6q24-TNDM, identifying paternal UPD6, paternal 6q24 duplications, or isolated maternal hypomethylation of the *PLAGL1*/*HYMAI* locus in patients with diabetes diagnosed in the first weeks of life and other coexisting disorders. These findings present additional grounds for the need for early combined sequencing and imprinting tests as genetic screenings to determine the etiology of neonatal diabetes. This will enable further medical care for these patients, with the need to monitor for diabetes recurrence and provide genetic counseling for entire families. However, due to the small sample size in our study and retrospective analysis, a prospective multicenter validation would provide a comprehensive picture of the disease.

## Figures and Tables

**Table 1:** Clinical characteristics of and molecular findings for five patients with 6q24-related TNDM. IUGR—intrauterine growth retardation; MS-MLPA—Methylation-specific Multiplex Ligation Probe Amplification; pat—paternal; patUPD6—paternal uniparental disomy; mo—months; yrs—years; F—female; M—male, ADHD—attention deficit hyperactivity disorder; SD—standard deviation; D—diabetes; GD—gestational diabetes; H—hyperglycemia; CS—cesarean section; pc—percentile.

Patient	Sex	Age at Genetic Diagnosis (Years)	Diabetes Diagnosis Age (Days)	Family History of Hyperglycemia/Diabetes	Maternal/ Obstetric History	IUGR	Gestation Age (Weeks)	Birth Weight (g)	MS-MLPA Analysis of 6q24	Follow-Up	Associated/ Neurodevelopmental Features **
P1	F	26	2	D—brother; H—father	CS due to risk of perinatal asphyxia	Yes	41	1950 [<3pc]	Paternal 6q24 duplication	Remission at 6 mo.; recurrence at 12 yrs	Autism; mild speech delay
P2	M	4	2	D—pat. grandfather; H—father	CS	Yes	34	1450 [<3pc]	patUPD6 or maternal hypomethylation	Remission at 3 mo.	Umbilical hernia
P3	M	1	3	D—Both grandmothers	CS due to risk of perinatal asphyxia and oligohydramnios	Yes	39	2340 [<3pc]	patUPD6 or maternal hypomethylation	Remission at 2 mo.	None
P4 *	M	12	14	D—pat. grandfather; H—mother	CS	No	40	2450 [3–10pc]	Paternal 6q24 duplication	Remission at 5 mo.	ADHD
P5	F	1.25	1	D—pat. grandmother; GD—mother	GD CS	Yes	29	1050 [<3pc]	patUPD6 or maternal hypomethylation	Remission at 5 mo.	Umbilical hernia; atrial septal defect; ↑ limb tone; ↓ abdominal tone
Average ± SD	-	8.85 ± 10.57	4.4 ± 5.1	-	-	-	36.6 ± 5.03	1848 ± 594	-	-	-

*—atypical case; **—data come from retrospective clinical reports.

**Table 2:** Comparative summary: Our cohort vs. published 6q24 TNDM cohorts. IUGR—Intrauterine growth restriction; SGA—small for gestational age; ADHD—attention deficit hyperactivity disorder; SD—standard deviation; NDM—neonatal diabetes mellitus.

Metric	This Study (Poland)	International Cohorts (Examples) [2,13]
Age at onset	All 5/5 within first 2 weeks (95% CI: 48–100%)	International cohort median ~day 1–4; typically within first week
IUGR/SGA	4/5 (80%) (95% CI: 28–99%)	Majority SGA; mean ~2.0 kg (~−2 to −2.5 SD)
Umbilical hernia	2/5 (40%) (95% CI: 5–85%)	~20–25%
Macroglossia	0/5 (0%) (95% CI: 0–45%)	~40–45%
Neurodevelopmental features	2/5 with diagnoses (autism; ADHD) (chart-based; no standardized testing)	Variable; not universally reported; more common in KATP-related NDM cohorts

**Table 3:** Recurrence risk and genetic counseling considerations by molecular mechanism of 6q24-related TNDM. MLID—multi-locus imprinting disturbance.

Molecular Mechanism	Typical Origin	Recurrence Risk	Recommended Counseling and Actions
Paternal UPD6	Sporadic (de novo)	Negligible (<1%)	Reassure family; no increased recurrence risk for siblings
Paternal 6q24 duplication	Inherited or de novo	Up to 50% if father is a carrier	Recommend parental MS-MLPA or microarray; inform of male-line transmission
Isolated maternal hypomethylation	Sporadic or rare familial	Low; rarely familial	Consider *ZFP57* sequencing; screen for MLID; counsel on low sibling risk

## Data Availability

The de-identified data that support the findings of this study are available from the corresponding author upon reasonable request.

## Abbreviations

The following abbreviations are used in this manuscript:

TNDM	Transient neonatal diabetes mellitus
PNDM	Permanent neonatal diabetes mellitus
MS-MLPA	Methylation-specific multiplex ligation-dependent probe amplification
MODY	Maturity-onset diabetes of the young
IUGR	Intrauterine growth restriction
tNGS	Targeted next-generation sequencing
UPD	Uniparental disomy
ADHD	Attention deficit hyperactivity disorder
DMR	Differentially methylated region
MLID	Multi-locus imprinting disturbance
NDM	Neonatal diabetes
MD	Monogenic diabetes
STR	Short tandem repeat
HbA1c	Glycated hemoglobin
SGA	Small for gestational age
SD	Standard deviation
pc	percentile
mtDNA	Mitochondrial DNA
